# Effect of oral urea ingestion on growth hormone levels in healthy adults - a secondary analysis of a randomized, double-blind, placebo-controlled cross-over trial

**DOI:** 10.1007/s11102-025-01577-2

**Published:** 2025-10-10

**Authors:** Friederike Riehle, Sven Lustenberger, Emanuel Christ, Sophie Monnerat, Cihan Atila, Mirjam Christ-Crain

**Affiliations:** 1 Department of Endocrinology, Diabetology and Metabolism, University Hospital Basel, Petersgraben 4, 4031 Basel, Switzerland; 2 Department of Clinical Research, University Hospital Basel, Basel, Switzerland

**Keywords:** Growth hormone, Growth hormone deficiency, Urea, Anterior pituitary stimulation test

## Abstract

**Purpose:**

Available stimulation tests for growth hormone (GH) deficiency are burdensome or require intravenous access, highlighting the need for accessible, oral options. Increasing plasma urea through oral ingestion may stimulate GH secretion via feedback regulation. In this analysis, we investigated the effect of oral urea on GH levels.

**Methods:**

This is a secondary analysis of a double-blind, randomized, placebo-controlled cross-over trial in healthy adults. They received a single weight-adapted dose of oral urea (0.5 g/kg body weight; 30–45 g) or placebo in random order. Serum GH was measured at baseline, 60 and 120 min. The main outcome was the difference in absolute GH levels after placebo and urea intake.

**Results:**

Of 22 healthy adults, 12 (55%) were female, with a median [IQR] age of 27 years [26, 32] and a body mass index of 23.3 kg/m^2^ [21.6, 25.8]. Before urea ingestion, baseline plasma urea was 4.4 mmol/L [3.5, 5.0], peaking after 60 min at 16.8 mmol/L [14.5, 18.0]. During the placebo visit, baseline urea levels were 4.3 mmol/L [3.7, 5.6] and remained stable during observation. Upon urea ingestion, median GH at baseline was 0.83 µg/l [0.28, 5.75], maximum GH levels were observed after 120 min at 1.00 µg/l [0.71, 2.34]. GH levels during placebo testing were 0.93 µg/l [0.38, 5.34] at baseline and 0.73 µg/l [0.18, 1.59] at 120 min (*p* = 0.08).

**Conclusion:**

Oral urea does not significantly stimulate GH secretion and cannot be used as a stimulation test for GH deficiency.

## Introduction

Diagnosing growth hormone deficiency (GHD) is important, as treatment improves quality of life, body composition and exercise capacity [1]. The diagnosis of GHD in adults relies on measurement of stimulated GH. Established stimulation tests are the insulin tolerance test (ITT), GH-releasing hormone (GHRH) - arginine test, glucagon stimulation test and Macimorelin. The ITT, GHRH-arginine, and glucagon test require the injection or infusion of the stimulating agent, and may therefore be burdensome for patients [2, 3]. Macimorelin, the sole oral option, is costly and unavailable in several countries, making other oral options for GH stimulation highly desirable [4].

Plasma urea, the end product of protein metabolism [5] rises with increased protein degradation [6]. GH promotes protein synthesis and lowers plasma urea by reducing urea production [7]. High levels of plasma urea might therefore stimulate GH secretion. The stimulatory effect of arginine, a key component of the urea cycle, on GH [8] further supports this hypothesis.

Urea may be administered orally – dissolved in a beverage – and is currently used to treat hyponatremia caused by the syndrome of inappropriate antidiuresis [9]. The use of urea is cost-effective and safe [10].

In this analysis, we investigated the potential of urea as an alternative stimulation test for GHD by assessing the effect of oral urea on GH secretion in healthy adults.

## Methods

This is a secondary analysis of a monocentric, randomized, double-blind, placebo-controlled cross-over trial in healthy adults investigating oral urea as a copeptin stimulation test for polyuria-polydipsia syndrome [10].

The study was conducted at the University Hospital Basel, Switzerland (June 2023-June 2024), approved by the local ethics committee (EKNZ 2023 − 00751), registered at ClinicalTrials.gov (NCT05890690), and conducted according to the Declaration of Helsinki. All participants gave written informed consent prior to any study procedures.

Twenty-two healthy individuals aged ≥ 18 years using no medication except hormonal contraception were included.

Exclusion criteria were evident disordered drinking habits and diuresis (polydipsia > 3 L/24 h, polyuria > 40-50mL/kg body weight/24 h), pregnancy, breastfeeding, allergies to components of the study drink, or recent investigational drug trial participation (within 30 days).

Subjects attended two morning study visits after overnight fasting and two-hours of fluid restriction to receive urea or placebo in random order, with a wash-out of at least 3 days. Participants were asked to abstain from heavy exercise, consumption of alcohol and nicotine in preparation for the study visit.

One weight-adapted dose of urea (0.5 g urea/kg body weight, 30–45 g) was dissolved in 200 ml water with lemon-lime flavored powder (0.4 g per g urea, containing maltodextrin and citric acid) to improve the bitter taste of urea. Both urea and lemon-lime flavor powder were manufactured by OMANDA AG, Switzerland.

The placebo drink, designed to mimic the taste of urea, contained bitter-tasting nutritional supplements (20 ml Ergytonyl^®^, 1 ml Carmol^®^, 1 ml Bitter Liebe^®^) and lemon-lime flavor in 200 ml water.

Unblinded staff members, otherwise not involved in the study prepared the study drinks, ensuring blinding of participants and investigators.

Baseline blood samples were collected ≥ 20 min after venous catheter insertion. Subjects then received the study drink to consume within 10 min, followed by 50 ml orange juice to mask the taste of urea.

Blood samples were taken 30, 60, 90, 120 and 150 min after study drink ingestion.

GH serum levels were measured at baseline, 60 and 120 min based on previously conducted studies, demonstrating GH peaks usually occur between 60 and 120 min and last about 60 minutes [2].

The central laboratory of the University Hospital Basel performed all laboratory analyses. Urea and glucose were measured immediately; samples for GH measurement were stored at − 80 °C until batch analysis and measured by electrochemiluminescence immunoassay (ECLIA, intra-assay variability coefficient: <1%, inter-assay variability coefficient: <4% [11] , Cobas8000, Roche Diagnostics GmbH, Germany).

Laboratory parameters over time were evaluated with summary statistics and visual representation of the data (box plots). Numeric data is presented as median with interquartile range (IQR), categorical data as frequency with percentage.

The main outcome was the absolute GH level difference after placebo and urea. P-values were calculated using Wilcoxon signed-rank test for paired samples to assess GH changes between urea and placebo intake. Statistical analyses were performed in R v4.4.3.

## Results

Of twenty-two healthy adults, 12 (55%) were female, median [IQR] age 27 years [26, 32], body mass index 23.3 kg/m^2^ [21.6, 25.8] (Table 1).

**Table 1:** Numerical variables are presented as median [IQR], categorical variables as n (%)

	Healthy adults (*n* = 22)	
Demographics		
Age (years)	27 [26, 32]	
Sex (female)	12 (55)	
Body Mass Index (kg/m^2^)	23.3 [21.6, 25.8]	
Test	Urea	Placebo
Vital signs		
Systolic blood pressure (mmHg)	113 [103, 123]	112 [103, 121]
Diastolic blood pressure (mmHg)	69 [62, 73]	63 [56, 73]
Heart rate (bpm)	61 [54, 69]	60 [54, 70]
Laboratory measurements		
Urea (mmol/L)	4.4 [3.5, 5.0]	4.3 [3.7, 5.6]
GH (µg/l)	0.83 [0.28, 5.75]	0.93 [0.38, 5.34]
Glucose (mmol/L)	4.7 [4.4, 4.8]	4.6 [4.5, 4.9]
Estimated glomerular filtration rate (ml/kg/1.73m^2^)	113 [102, 123]	116 [4.5, 4.9]
Creatinine (µmol/l)	68 [64, 76]	68 [63, 73]
Osmolality (mOsm/kg)	292 [288, 294]	293 [290, 295]

With urea ingestion, baseline plasma urea was 4.4 mmol/L [3.5, 5.0], peaking at 16.8 mmol/L [14.5, 18.0] after 60 min and remaining stable at 16.6 mmol/L [14.4, 17.8] after 120 min.

During placebo visits, baseline urea was 4.3 mmol/L [3.7, 5.6], 4.0 mmol/L [3.6, 5.4] after 60 min and 4.1 mmol/L [3.5, 5.1] after 120 min.

Baseline GH was 0.83 µg/l [0.28, 5.75] before urea ingestion and 0.93 µg/l [0.38, 5.34] before placebo ingestion (*p* = 0.80). At 60 min, GH decreased to 0.38 µg/l [0.20, 2.46] after urea and to 0.38 µg/l [0.16, 1.51] after placebo (*p* = 0.18). After 120 min, GH was 1.00 µg/l [0.71, 2.34] after urea and 0.73 µg/l [0.18, 1.59] after placebo (*p* = 0.08).

Before urea ingestion, baseline glucose was 4.7 mmol/l [4.4, 4.8], peaked after 30 min at 5.9 mmol/L [5.4, 6.4], decreased to 5.3 mmol/l [4.9, 5.6] after 60 min and to 4.7 mmol/l [4.3, 4.9] after 120 min.

**Fig. 1:**
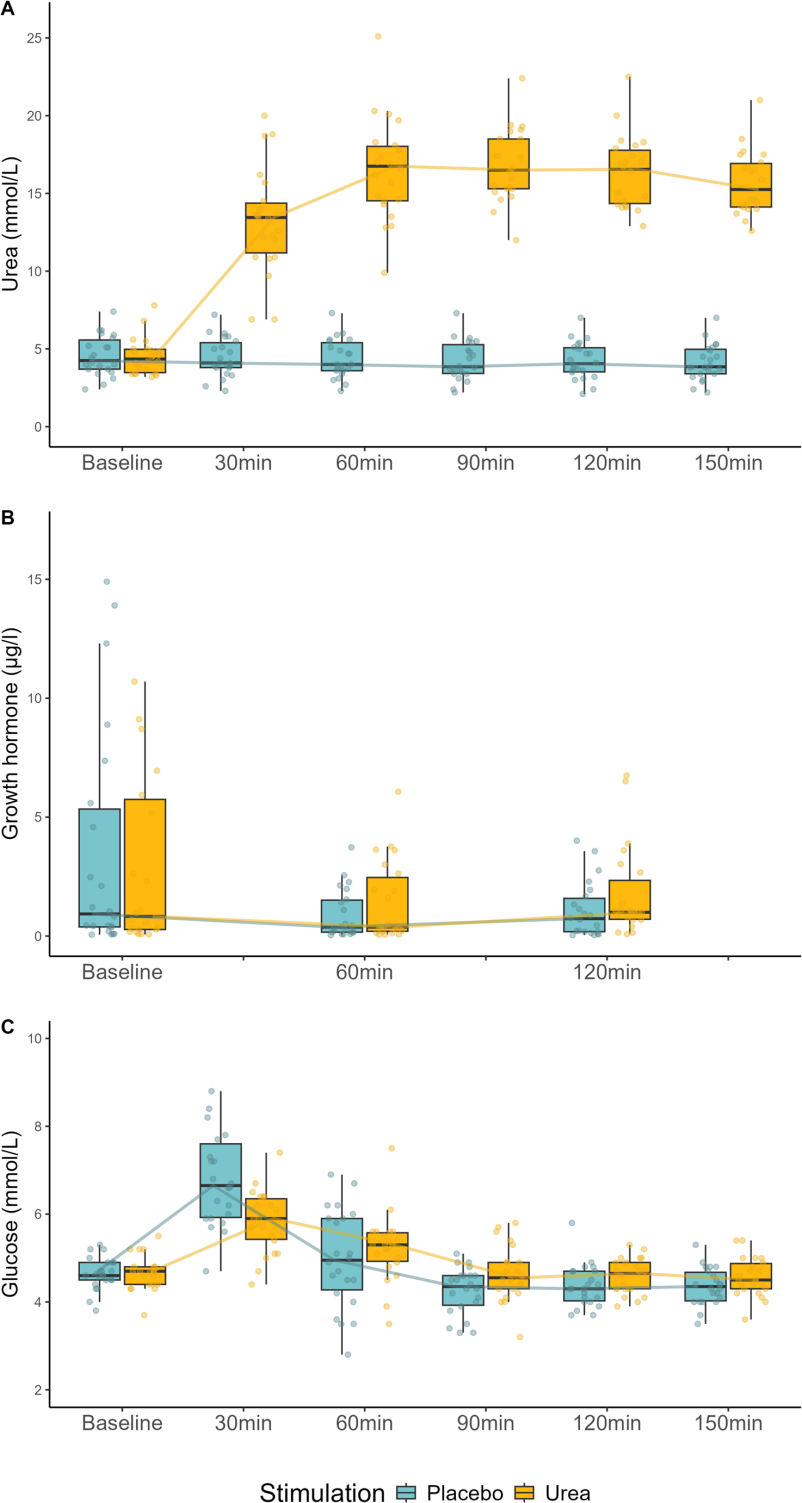
Serum levels over time of urea, GH and glucose after urea or placebo ingestion. Boxes span the interquartile range (IQR); the thick horizontal line is the median. Whiskers represent the most extreme values lying within the box edges and 1.5 times the interquartile range (IQR). All other values are outliers and plotted as individual points

Before placebo ingestion, baseline glucose was 4.6 mmol/l [4.5, 4.9], peaked after 30 min at 6.7 mmol/L [5.9, 7.6], decreased to 5.0 mmol/l [4.3, 5.9] after 60 min and to 4.3 mmol/l [4.0, 4.7] after 120 min (Fig. 1).

## Discussion

Despite significantly increased plasma urea after urea intake, no significant difference in GH levels was observed between urea and placebo ingestion.

Absolute GH values declined slightly following both placebo and urea ingestion, with a tendency to increase slightly by 120 min. Oral agents may result in a delayed GH peak compared to intravenously administered stimuli and a GH peak later than 120 min cannot be fully excluded. However, given the prompt rise in GH upon stimulation with Macimorelin [12] we consider this unlikely.

Unlike arginine infusion, urea appears not to stimulate GH secretion. Arginine, converted to urea in the urea cycle, increases GH secretion. Although the exact mechanism remains uncertain, arginine is postulated to suppress the tonic somatostatin secretion, which de-inhibits GH stimulation via α-adrenergic and serotonergic signaling [13]. Our study suggests urea is not involved in this pathway, despite its close metabolic relation to arginine.

In contrast, GH’s influence on urea is well established; numerous studies showed GH secretion to decrease urea production [14].

This study’s strength is its robust methodological design, minimizing biases and confounders. Study visits occurred in the morning after overnight fasting aligning with GH’s circadian secretion pattern [15, 16].

As GH is a stress hormone, baseline values may be falsely elevated immediately after venous catheter placement [17]. To minimize this, baseline samples were obtained ≥ 20 min after catheter placement.

As a limitation, glucose contained in the orange juice used for masking the taste of urea may have influenced GH levels: Blood glucose levels were elevated shortly after consumption, with a peak after 30 min. Ingestion of glucose is known to suppress GH secretion [18]. However, given that GH levels were comparable after urea and placebo, a relevant increase in GH following ingestion of urea is highly unlikely – even without juice.

To conclude, oral urea does not stimulate GH in healthy adults and cannot be utilized as a novel stimulation test to diagnose GHD.

## Data Availability

No datasets were generated or analysed during the current study.
